# Multivalency regulates activity in an intrinsically disordered transcription factor

**DOI:** 10.7554/eLife.40684

**Published:** 2018-08-03

**Authors:** Sarah Clark, Janette B Myers, Ashleigh King, Radovan Fiala, Jiri Novacek, Grant Pearce, Jörg Heierhorst, Steve L Reichow, Elisar J Barbar

Clark S, Myers JB, King A, Fiala R, Novacek J, Pearce G, Heierhorst J, Reichow SL, Barbar EJ. 2018. Multivalency regulates activity in an intrinsically disordered transcription factor. *eLife* **7**:e36258. doi: 10.7554/eLife.36258.

Published 1, May 2018

After publication we became aware of three citations that should be included in the Materials and Methods.

The (Motáčková et al., 2010; Nováček et al., 2011) citation In the Materials and Methods subsection NMR experiments should also include Kazimierczuk et al., 2010.

The new citation is shown below:

Kazimierczuk, K., Zawadzka-Kazimierczuk, A., Kozminski, W. 2010. Non-uniform frequency domain for optimal exploitation of non-uniform sampling. *J Magn Reson,* 205(2), 286-292. doi:10.1016/j.jmr.2010.05.012

The other two citations refer to the processing of non-uniformly sampled five-dimensional HN(CA)CONH and HabCabCONH spectra. The citations should be placed within the text as follows:

“All two-dimensional spectra and the three-dimensional HNCO spectra were processed using TopSpin (Bruker Biosciences; RRID:SCR_014227), and the non- uniformly sampled five-dimensional HN(CA)CONH and HabCabCONH spectra were processed with Sparse Multidimensional Fourier Transform (Kazimierczuk et al., 2009; Stanek et al., 2010) (the software for data processing is available online at the Warsaw University Laboratory (nmr.cent3.uw.edu.pl/software)).”

The citations are shown below:

Kazimierczuk, K., Zawadzka, A., Kozminski, W. 2009. Narrow peaks and high dimensionalities: exploiting the advantages of random sampling. *J Magn Reson,* 197(2), 219-228. doi:10.1016/j.jmr.2009.01.003

Stanek, J., Kozminski, W. (2010). Iterative algorithm of discrete Fourier transform for processing randomly sampled NMR data sets. *J Biomol NMR,* 47(1), 65-77. doi:10.1007/s10858-010-9411-2

The article has been corrected accordingly.

