# Supplementary material for: Multivalency regulates activity in an intrinsically disordered transcription factor
Source: eLife. 2018 May 1;7:e36258. doi: 10.7554/eLife.36258 (PMC5963919; doi:10.7554/eLife.36258)
Supplement: Figure 1—source data 1. [file elife-36258-fig1-data1.docx]

| PDB ID | Protein name | Motif sequence |
| --- | --- | --- |
| 4QH7 | Anastral spindle 2 | ASSTTGTQCD |
| 4QH8 | Anastral spindle 2 | lTICAGTQTD |
| 2P2T | Cytoplasmic dynein intermediate chain | LVYTKQTQTT |
| 2XQQ | Echinoderm microtubule-associated protein-like 3 | SLVSRGTQTE |
| 5E0M | Chica | WSRSTTTQTD |
| 5E0L | Chica | KAIDAATQTE |
| 5D07 | Myosin Va | PKDDKNTMTD |
| 1CMI | Nitric oxide synthase, brain | EMKDTGIQVD |
| 3ZKE | Nek9 | GMHSKGTQTA |
| 3E2B | Swallow | ATSAKATQTDf |
| 3DVT | PAK1 | PTRDVATSPI |
