# Supplementary material for: Multivalency regulates activity in an intrinsically disordered transcription factor
Source: eLife. 2018 May 1;7:e36258. doi: 10.7554/eLife.36258 (PMC5963919; doi:10.7554/eLife.36258)
Supplement: Figure 2—source data 1. — Cα and Cβ chemical shifts are listed for Figure 2e, R1 and R2 relaxation rates for Figure 2f, and Iunsat/Isat values for Figure 2g. [file elife-36258-fig2-data1.docx]

| **Figure 2e** |  | |  | |  |  |  |
| --- | --- | --- | --- | --- | --- | --- | --- |
|  | **Measured values** | | **Random coil values** | |  |  |  |
| **Residue Number** | Cα | Cβ | Cα | Cβ | ΔCα | ΔCβ | ΔCα - ΔCβ |
| 241 | 55.4 | 32.5 | 55.5 | 32.8 | 0.2 | 0.3 | -0.1 |
| 242 | 54.7 | 41.2 | 54.3 | 40.7 | -0.4 | -0.5 | 0.2 |
| 243 | 62.7 | 32.7 | 62.8 | 32.6 | 0.1 | -0.1 | 0.2 |
| 244 | 58.8 | 63.7 | 58.5 | 63.5 | -0.3 | -0.1 | -0.1 |
| 245 | 0.0 | 0.0 | 57.9 | 38.7 | 0.0 | 0.0 | 0.0 |
| 246 | 52.9 | 19.1 | 52.2 | 19.0 | -0.7 | -0.1 | -0.5 |
| 247 | 55.7 | 42.3 | 55.4 | 42.3 | -0.3 | -0.1 | -0.3 |
| 248 | 0.0 | 0.0 | 56.6 | 29.8 | 0.0 | 0.0 | 0.0 |
| 249 | 55.8 | 32.7 | 55.5 | 32.6 | -0.3 | -0.1 | -0.2 |
| 250 | 59.0 | 63.7 | 58.5 | 63.7 | -0.4 | -0.1 | -0.4 |
| 251 | 58.8 | 63.7 | 58.6 | 63.8 | -0.2 | 0.1 | -0.3 |
| 252 | 56.1 | 29.4 | 56.1 | 29.3 | 0.0 | -0.1 | 0.1 |
| 253 | 56.4 | 33.0 | 56.4 | 32.8 | 0.0 | -0.2 | 0.1 |
| 254 | 55.2 | 42.5 | 55.3 | 42.3 | 0.1 | -0.2 | 0.3 |
| 255 | 54.5 | 40.9 | 54.4 | 40.8 | -0.1 | -0.1 | 0.0 |
| 256 | 55.6 | 32.9 | 55.6 | 32.7 | 0.0 | -0.3 | 0.3 |
| 257 | 56.9 | 30.3 | 56.8 | 30.0 | -0.1 | -0.2 | 0.1 |
| 258 | 62.1 | 69.9 | 62.1 | 70.1 | 0.0 | 0.2 | -0.2 |
| 259 | 55.8 | 29.6 | 55.9 | 29.5 | 0.1 | -0.1 | 0.2 |
| 260 | 61.9 | 70.1 | 62.2 | 69.8 | 0.2 | -0.3 | 0.5 |
| 261 | 56.6 | 30.4 | 56.7 | 30.4 | 0.1 | 0.0 | 0.1 |
| 262 | 56.7 | 30.4 | 56.9 | 30.2 | 0.1 | -0.2 | 0.3 |
| 263 | 54.8 | 41.3 | 54.7 | 41.0 | -0.1 | -0.3 | 0.2 |
| 264 | 54.6 | 41.0 | 54.5 | 40.6 | -0.1 | -0.4 | 0.2 |
| 265 | 55.8 | 41.9 | 55.6 | 41.9 | -0.2 | 0.0 | -0.2 |
| 266 | 53.9 | 39.0 | 53.3 | 38.5 | -0.5 | -0.5 | 0.0 |
| 267 | 56.8 | 30.4 | 56.7 | 30.3 | -0.2 | -0.1 | -0.1 |
| 268 | 0.0 | 0.0 | 61.2 | 38.5 | 0.0 | 0.0 | 0.0 |
| 269 | 56.0 | 30.9 | 56.1 | 30.7 | 0.1 | -0.3 | 0.3 |
| 270 | 53.4 | 39.1 | 53.3 | 38.7 | -0.1 | -0.3 | 0.2 |
| 271 | 56.7 | 30.4 | 56.9 | 30.2 | 0.2 | -0.2 | 0.4 |
| 272 | 0.0 | 0.0 | 62.4 | 32.6 | 0.0 | 0.0 | 0.0 |
| 273 | 0.0 | 0.0 | 54.8 | 42.4 | 0.0 | 0.0 | 0.0 |
| 274 | 0.0 | 0.0 | 50.4 | 18.4 | 0.0 | 0.0 | 0.0 |
| 275 | 63.1 | 32.1 | 63.0 | 32.2 | -0.1 | 0.0 | -0.1 |
| 276 | 55.2 | 42.2 | 55.1 | 42.3 | -0.1 | 0.1 | -0.2 |
| 277 | 55.1 | 42.2 | 55.1 | 42.2 | -0.1 | 0.0 | -0.1 |
| 278 | 0.0 | 0.0 | 56.1 | 30.9 | 0.0 | 0.0 | 0.0 |
| 279 | 0.0 | 0.0 | 54.2 | 41.0 | 0.0 | 0.0 | 0.0 |
| 280 | 0.0 | 0.0 | 61.4 | 38.5 | 0.0 | 0.0 | 0.0 |
| 281 | 0.0 | 0.0 | 56.6 | 30.0 | 0.0 | 0.0 | 0.0 |
| 282 | 62.1 | 69.8 | 62.1 | 70.1 | 0.0 | 0.3 | -0.3 |
| 283 | 55.6 | 29.6 | 55.7 | 29.7 | 0.1 | 0.1 | 0.1 |
| 284 | 0.0 | 0.0 | 59.9 | 69.2 | 0.0 | 0.0 | 0.0 |
| 285 | 63.4 | 32.2 | 63.2 | 32.4 | -0.2 | 0.2 | -0.4 |
| 286 | 54.4 | 41.2 | 54.5 | 41.2 | 0.1 | 0.0 | 0.1 |
| 287 | 61.9 | 69.6 | 62.0 | 69.8 | 0.2 | 0.2 | 0.0 |
| 288 | 56.8 | 30.7 | 56.5 | 30.8 | -0.4 | 0.1 | -0.5 |
| 289 | 45.2 | 0.0 | 45.2 | 0 | 0.0 | 0.0 | 0.0 |
| 290 | 54.2 | 41.1 | 54.4 | 40.9 | 0.1 | -0.2 | 0.3 |
| 291 | 61.7 | 38.7 | 61.5 | 38.4 | -0.1 | -0.3 | 0.1 |
| 292 | 45.4 | 0.0 | 45.4 | 0 | 0.0 | 0.0 | 0.0 |
| 293 | 62.0 | 69.9 | 62.1 | 69.9 | 0.1 | 0.0 | 0.1 |
| 294 | 55.6 | 32.9 | 55.5 | 32.9 | -0.1 | 0.0 | -0.1 |
| 295 | 61.7 | 70.0 | 62.2 | 69.8 | 0.5 | -0.1 | 0.6 |
| 296 | 54.4 | 41.3 | 54.8 | 41.2 | 0.4 | -0.1 | 0.5 |
| 297 | 54.3 | 41.3 | 54.5 | 40.9 | 0.3 | -0.4 | 0.7 |
| 298 | 0.0 | 0.0 | 55.7 | 38.6 | 0.0 | 0.0 | 0.0 |
| 299 | 63.1 | 32.1 | 63.0 | 32.1 | -0.1 | 0.0 | -0.1 |
| 300 | 56.6 | 30.5 | 56.8 | 30.2 | 0.2 | -0.3 | 0.5 |
| 301 | 56.5 | 30.4 | 56.7 | 30.4 | 0.2 | 0.0 | 0.3 |
| 302 | 55.4 | 30.0 | 55.8 | 29.4 | 0.4 | -0.5 | 0.9 |
| 303 | 0.0 | 0.0 | 54.5 | 29.6 | 0.0 | 0.0 | 0.0 |
| 304 | 63.1 | 32.1 | 63.0 | 32.2 | -0.1 | 0.1 | -0.2 |
| 305 | 0.0 | 0.0 | 62.2 | 32.9 | 0.0 | 0.0 | 0.0 |
| 306 | 52.3 | 19.3 | 52.2 | 19.2 | -0.1 | -0.1 | -0.1 |
| 307 | 0.0 | 0.0 | 62.6 | 32.6 | 0.0 | 0.0 | 0.0 |
| 308 | 45.2 | 0.0 | 45.3 | **.*** | 0.0 | 0.0 | 0.0 |
| 309 | 58.5 | 63.9 | 58.7 | 63.7 | 0.2 | -0.2 | 0.4 |
| 310 | 55.7 | 29.8 | 55.9 | 29.5 | 0.1 | -0.3 | 0.4 |
| 311 | 58.0 | 39.7 | 57.7 | 39.5 | -0.2 | -0.2 | 0.0 |
| 312 | 0.0 | 0.0 | 55.6 | 29.7 | 0.0 | 0.0 | 0.0 |
| 313 | 52.4 | 19.3 | 52.8 | 18.7 | 0.4 | -0.6 | 1.0 |
| 314 | 58.2 | 38.9 | 57.9 | 38.7 | -0.3 | -0.2 | -0.1 |
| 315 | 57.9 | 64.1 | 58.2 | 63.9 | 0.4 | -0.2 | 0.6 |
| 316 | 57.0 | 30.2 | 56.8 | 30.1 | -0.1 | -0.2 | 0.0 |
| 317 | 0.0 | 0.0 | 62.0 | 70.0 | 0.0 | 0.0 | 0.0 |
| 318 | 0.0 | 0.0 | 54.5 | 29.8 | 0.0 | 0.0 | 0.0 |
| 319 | 63.3 | 32.1 | 63.3 | 32.0 | 0.0 | -0.1 | 0.2 |
| 320 | 55.4 | 33.0 | 55.5 | 32.7 | 0.0 | -0.3 | 0.3 |
| 321 | 57.8 | 39.9 | 57.9 | 39.6 | 0.1 | -0.3 | 0.4 |
| 322 | 54.1 | 41.3 | 54.1 | 40.9 | 0.0 | -0.4 | 0.4 |
| 323 | 55.9 | 42.2 | 55.6 | 42.2 | -0.4 | 0.0 | -0.4 |
| 324 | 56.5 | 29.1 | 56.0 | 28.9 | -0.5 | -0.3 | -0.3 |
| 325 | 62.5 | 69.7 | 62.1 | 70.0 | -0.4 | 0.3 | -0.6 |
| 326 | 0.0 | 0.0 | 58.4 | 63.9 | 0.0 | 0.0 | 0.0 |
| 327 | 53.0 | 19.1 | 52.8 | 19.0 | -0.3 | -0.1 | -0.2 |
| 328 | 55.9 | 29.7 | 55.9 | 29.6 | 0.0 | 0.0 | 0.0 |
| 329 | 55.7 | 32.9 | 55.5 | 32.7 | -0.2 | -0.1 | 0.0 |
| 330 | 57.9 | 38.8 | 57.9 | 38.6 | 0.0 | -0.3 | 0.2 |
| 331 | 61.8 | 70.0 | 61.8 | 70.1 | 0.0 | 0.1 | -0.1 |
| 332 | 56.0 | 29.7 | 56.0 | 29.6 | 0.0 | -0.2 | 0.2 |
| 333 | 61.9 | 70.1 | 62.2 | 69.8 | 0.3 | -0.3 | 0.5 |
| 334 | 58.6 | 28.2 | 58.6 | 29.9 | 0.0 | 1.7 | -1.0 |
| 335 | 55.0 | 41.2 | 54.9 | 41.0 | -0.1 | -0.2 | 0.1 |
| 336 | 54.9 | 41.0 | 54.7 | 40.6 | -0.2 | -0.4 | 0.2 |
| 337 | 55.4 | 42.2 | 55.5 | 41.9 | 0.1 | -0.2 | 0.3 |
| 338 | 58.4 | 39.3 | 57.7 | 39.2 | -0.7 | -0.1 | -0.5 |
| 339 | 56.9 | 30.5 | 56.5 | 30.4 | -0.3 | -0.1 | -0.2 |
| 340 | 57.1 | 30.0 | 56.6 | 30.0 | -0.5 | -0.1 | -0.4 |
| 341 | 55.6 | 42.3 | 55.6 | 42.3 | 0.0 | 0.0 | 0.0 |
| 342 | 45.7 | 0.0 | 45.2 | 0 | 0.0 | 0.0 | 0.0 |
| 343 | 55.3 | 42.4 | 55.3 | 42.4 | 0.0 | 0.0 | 0.0 |
| 344 | 58.8 | 63.7 | 58.4 | 63.6 | -0.3 | -0.1 | -0.2 |
| 345 | 56.1 | 29.7 | 55.5 | 29.8 | -0.5 | 0.1 | -0.6 |
| 346 | 0.0 | 0.0 | 61.3 | 38.8 | 0.0 | 0.0 | 0.0 |
| 347 | 56.0 | 29.5 | 55.8 | 29.3 | -0.2 | -0.2 | 0.0 |
| 348 | 62.2 | 69.8 | 62.1 | 70.0 | -0.1 | 0.2 | -0.3 |
| 349 | 55.9 | 29.5 | 55.9 | 29.5 | 0.0 | 0.0 | 0.0 |
| 350 | 61.9 | 69.9 | 62.2 | 69.7 | 0.3 | -0.2 | 0.5 |
| 351 | 0.0 | 0.0 | 55.8 | 29.9 | 0.0 | 0.0 | 0.0 |
| 352 | 0.0 | 0.0 | 55.2 | 28.6 | 0.0 | 0.0 | 0.0 |
| 353 | 0.0 | 0.0 | 63.1 | 32.2 | 0.0 | 0.0 | 0.0 |
| 354 | 54.8 | 41.2 | 54.9 | 40.9 | 0.0 | -0.3 | 0.4 |
| 355 | 45.7 | 0.0 | 45.5 | 0.0 | -0.2 |  | -0.2 |
| 356 | 55.4 | 42.3 | 55.5 | 42.1 | 0.1 | -0.3 | 0.4 |
| 357 | 57.9 | 38.7 | 57.9 | 38.4 | 0.0 | -0.3 | 0.3 |
| 358 | 53.3 | 38.8 | 53.0 | 38.9 | -0.3 | 0.1 | -0.4 |
| 359 | 62.3 | 69.6 | 62.4 | 69.8 | 0.1 | 0.1 | -0.1 |
| 360 | 0.0 | 0.0 | 56.1 | 29.5 | 0.0 | 0.0 | 0.0 |
| 361 | 0.0 | 0.0 | 55.8 | 29.8 | 0.0 | 0.0 | 0.0 |
| 362 | 62.1 | 69.8 | 62.1 | 70.0 | 0.0 | 0.2 | -0.2 |
| 363 | 56.0 | 29.8 | 56.0 | 29.6 | 0.0 | -0.2 | 0.2 |
| 364 | 0.0 | 0.0 | 62.2 | 69.8 | 0.0 | 0.0 | 0.0 |
| 365 | 58.7 | 28.2 | 58.6 | 29.9 | 0.0 | 1.8 | -1.0 |
| 366 | 54.9 | 41.2 | 54.7 | 41.0 | -0.3 | -0.2 | -0.1 |
| 367 | 56.8 | 30.4 | 56.7 | 30.1 | -0.1 | -0.3 | 0.2 |
| 368 | 0.0 | 0.0 | 61.2 | 38.4 | 0.0 | 0.0 | 0.0 |
| 369 | 55.3 | 32.9 | 55.4 | 32.7 | 0.1 | -0.2 | 0.3 |
| 370 | 54.7 | 41.3 | 54.6 | 41.0 | -0.1 | -0.3 | 0.3 |
| 371 | 56.7 | 30.4 | 56.9 | 29.9 | 0.1 | -0.5 | 0.7 |
| 372 | 55.1 | 42.5 | 55.2 | 42.2 | 0.1 | -0.3 | 0.4 |
| 373 | 0.0 | 0.0 | 55.4 | 38.7 | 0.0 | 0.0 | 0.0 |
| 374 | 63.4 | 32.2 | 63.0 | 32.4 | -0.5 | 0.2 | -0.6 |
| 375 | 54.7 | 41.0 | 54.8 | 40.8 | 0.2 | -0.2 | 0.4 |
| 376 | 53.4 | 38.6 | 53.5 | 38.5 | 0.1 | -0.1 | 0.2 |
| 377 | 58.6 | 39.3 | 58.1 | 39.1 | -0.5 | -0.3 | -0.3 |
| 378 | 56.0 | 29.3 | 55.8 | 29.4 | -0.2 | 0.0 | -0.2 |
| 379 | 58.8 | 63.8 | 58.6 | 63.7 | -0.2 | 0.0 | -0.1 |
| 380 | 62.0 | 69.7 | 62.2 | 69.8 | 0.1 | 0.0 | 0.1 |
| 381 | 58.9 | 27.9 | 58.6 | 29.9 | -0.3 | 2.0 | -1.0 |
| 382 | 62.3 | 69.6 | 62.3 | 69.9 | 0.0 | 0.3 | -0.4 |
| 383 | 56.0 | 29.5 | 56.0 | 29.7 | -0.1 | 0.2 | -0.3 |
| 384 | 62.2 | 69.7 | 62.1 | 69.7 | -0.1 | -0.1 | 0.0 |
| 385 | 56.0 | 31.0 | 56.6 | 30.8 | 0.5 | -0.2 | 0.7 |
| 386 | 57.3 | 29.6 | 57.2 | 29.1 | -0.1 | -0.6 | 0.5 |
| 387 | 0.0 | 0.0 | 55.3 | 42.4 | 0.0 | 0.0 | 0.0 |
| 388 | 0.0 | 0.0 | 54.6 | 40.7 | 0.0 | 0.0 | 0.0 |

| **Figure 2f** | | | | | | |
| --- | --- | --- | --- | --- | --- | --- |
| **Residue number** | **R_1_** | **R_1_ error** | **R_2_** | **R_2_ error** | **R_2_/R_1_** | **R_2_/R_1_ error** |
| 241 | 1.152 | 0.058 | 1.773 | 0.026 | 1.539 | 0.076 |
| 242 | 1.398 | 0.081 | 2.262 | 0.020 | 1.619 | 0.029 |
| 243 | 1.529 | 0.110 | 3.128 | 0.015 | 2.046 | 0.033 |
| 244 | 1.640 | 0.143 | 3.309 | 0.030 | 2.017 | 0.026 |
| 245 | 1.785 | 0.186 | 3.232 | 0.024 | 1.810 | 0.025 |
| 246 | 1.726 | 0.165 | 3.721 | 0.022 | 2.156 | 0.034 |
| 247 | 1.686 | 0.155 | 3.544 | 0.009 | 2.102 | 0.019 |
| 248 | 1.717 | 0.162 | 3.690 | 0.019 | 2.149 | 0.033 |
| 249 | 1.563 | 0.118 | 4.086 | 0.008 | 2.614 | 0.041 |
| 250 | 1.550 | 0.130 | 3.912 | 0.048 | 2.524 | 0.076 |
| 251 | 1.530 | 0.126 | 3.809 | 0.030 | 2.489 | 0.086 |
| 252 | 1.631 | 0.156 | 4.070 | 0.016 | 2.496 | 0.112 |
| 253 | 1.677 | 0.156 | 3.557 | 0.020 | 2.121 | 0.027 |
| 254 | 1.593 | 0.126 | 3.599 | 0.008 | 2.260 | 0.057 |
| 255 | 1.563 | 0.118 | 4.037 | 0.006 | 2.582 | 0.041 |
| 256 | 1.543 | 0.117 | 3.558 | 0.013 | 2.306 | 0.023 |
| 257 | 1.556 | 0.119 | 4.196 | 0.013 | 2.697 | 0.058 |
| 258 | 1.531 | 0.118 | 4.431 | 0.013 | 2.894 | 0.062 |
| 259 | 1.574 | 0.123 | 3.614 | 0.015 | 2.296 | 0.045 |
| 260 | 1.473 | 0.096 | 3.884 | 0.012 | 2.637 | 0.044 |
| 261 | 1.523 | 0.108 | 4.103 | 0.007 | 2.695 | 0.042 |
| 262 | 1.539 | 0.115 | 4.464 | 0.013 | 2.901 | 0.041 |
| 263 | 1.584 | 0.126 | 4.443 | 0.012 | 2.805 | 0.030 |
| 264 | 1.563 | 0.118 | 4.086 | 0.008 | 2.614 | 0.041 |
| 265 | 1.546 | 0.114 | 5.241 | 0.010 | 3.390 | 0.064 |
| 266 | 1.593 | 0.125 | 5.098 | 0.013 | 3.200 | 0.069 |
| 267 | 1.532 | 0.111 | 4.724 | 0.015 | 3.084 | 0.052 |
| 268 | 1.520 | 0.109 | 5.345 | 0.017 | 3.517 | 0.086 |
| 269 | 1.602 | 0.127 | 5.283 | 0.016 | 3.298 | 0.071 |
| 270 | 1.522 | 0.115 | 4.867 | 0.017 | 3.197 | 0.062 |
| 271 | 1.429 | 0.087 | 4.802 | 0.013 | 3.360 | 0.033 |
| 272 | 1.454 | 0.088 | 4.960 | 0.014 | 3.411 | 0.092 |
| 273 |  |  |  |  |  |  |
| 274 |  |  |  |  |  |  |
| 275 |  |  |  |  |  |  |
| 276 | 1.441 | 0.088 | 5.399 | 0.013 | 3.746 | 0.072 |
| 277 | 1.460 | 0.091 | 4.723 | 0.007 | 3.235 | 0.057 |
| 278 | 1.525 | 0.117 | 5.038 | 0.020 | 3.303 | 0.054 |
| 279 | 1.584 | 0.126 | 4.443 | 0.012 | 2.805 | 0.030 |
| 280 |  |  | 0.000 |  |  |  |
| 281 |  |  | 0.000 |  |  |  |
| 282 | 1.536 | 0.119 | 4.620 | 0.014 | 3.009 | 0.061 |
| 283 | 1.476 | 0.101 | 4.344 | 0.012 | 2.943 | 0.041 |
| 284 | 1.457 | 0.100 | 4.196 | 0.023 | 2.879 | 0.059 |
| 285 |  |  | 0.000 |  |  |  |
| 286 | 1.540 | 0.116 | 4.163 | 0.011 | 2.702 | 0.022 |
| 287 | 1.500 | 0.108 | 4.009 | 0.023 | 2.672 | 0.073 |
| 288 | 1.678 | 0.156 | 4.360 | 0.028 | 2.599 | 0.043 |
| 289 | 1.533 | 0.120 | 3.705 | 0.026 | 2.416 | 0.047 |
| 290 | 1.543 | 0.117 | 3.658 | 0.020 | 2.370 | 0.026 |
| 291 | 1.463 | 0.094 | 3.534 | 0.011 | 2.415 | 0.039 |
| 292 | 1.575 | 0.122 | 3.873 | 0.031 | 2.459 | 0.043 |
| 293 | 1.524 | 0.111 | 3.270 | 0.019 | 2.146 | 0.020 |
| 294 | 1.548 | 0.120 | 3.450 | 0.018 | 2.229 | 0.045 |
| 295 | 1.454 | 0.091 | 3.746 | 0.015 | 2.576 | 0.036 |
| 296 | 1.556 | 0.124 | 3.977 | 0.010 | 2.557 | 0.052 |
| 297 | 1.510 | 0.106 | 3.848 | 0.021 | 2.548 | 0.047 |
| 298 | 1.427 | 0.085 | 4.683 | 0.031 | 3.282 | 0.052 |
| 299 |  |  | 0.000 |  |  |  |
| 300 | 1.523 | 0.110 | 4.387 | 0.017 | 2.881 | 0.057 |
| 301 | 1.526 | 0.111 | 4.720 | 0.010 | 3.094 | 0.053 |
| 302 | 1.454 | 0.092 | 5.049 | 0.010 | 3.472 | 0.046 |
| 303 | 1.492 | 0.105 | 4.814 | 0.010 | 3.226 | 0.072 |
| 304 |  |  | 0.000 |  |  |  |
| 305 | 1.460 | 0.092 | 4.806 | 0.024 | 3.292 | 0.053 |
| 306 | 1.487 | 0.101 | 4.640 | 0.029 | 3.119 | 0.045 |
| 307 | 1.332 | 0.067 | 4.257 | 0.011 | 3.195 | 0.055 |
| 308 | 1.420 | 0.102 | 4.244 | 0.107 | 2.989 | 0.171 |
| 309 | 1.368 | 0.108 | 4.340 | 0.023 | 3.172 | 0.226 |
| 310 | 1.261 | 0.089 | 4.847 | 0.119 | 3.843 | 0.588 |
| 311 | 1.457 | 0.118 | 4.998 | 0.017 | 3.430 | 0.178 |
| 312 | 1.631 | 0.156 | 4.070 | 0.016 | 2.496 | 0.112 |
| 313 | 1.430 | 0.105 | 5.329 | 0.024 | 3.727 | 0.139 |
| 314 | 1.486 | 0.104 | 5.349 | 0.028 | 3.600 | 0.060 |
| 315 | 1.515 | 0.114 | 5.645 | 0.020 | 3.727 | 0.075 |
| 316 | 1.519 | 0.111 | 5.506 | 0.020 | 3.625 | 0.068 |
| 317 |  |  | 0.000 |  |  |  |
| 318 |  |  | 0.000 |  |  |  |
| 319 |  |  | 0.000 |  |  |  |
| 320 | 1.559 | 0.120 | 5.779 | 0.023 | 3.708 | 0.045 |
| 321 | 1.355 | 0.074 | 5.908 | 0.019 | 4.361 | 0.086 |
| 322 | 1.493 | 0.096 | 5.743 | 0.014 | 3.847 | 0.138 |
| 323 | 1.475 | 0.097 | 5.765 | 0.015 | 3.908 | 0.058 |
| 324 | 1.554 | 0.121 | 6.187 | 0.021 | 3.982 | 0.083 |
| 325 | 1.484 | 0.105 | 5.707 | 0.091 | 3.847 | 0.138 |
| 326 | 1.474 | 0.111 | 5.556 | 0.026 | 3.770 | 0.145 |
| 327 | 1.488 | 0.114 | 5.678 | 0.024 | 3.815 | 0.120 |
| 328 | 1.383 | 0.102 | 5.897 | 0.020 | 4.263 | 0.203 |
| 329 | 1.478 | 0.107 | 5.666 | 0.014 | 3.833 | 0.071 |
| 330 | 1.466 | 0.092 | 6.124 | 0.029 | 4.176 | 0.105 |
| 331 | 1.450 | 0.092 | 6.259 | 0.034 | 4.317 | 0.095 |
| 332 | 1.480 | 0.103 | 6.239 | 0.036 | 4.215 | 0.071 |
| 333 | 1.472 | 0.102 | 5.524 | 0.020 | 3.753 | 0.087 |
| 334 | 1.415 | 0.094 | 5.355 | 0.038 | 3.784 | 0.122 |
| 335 | 1.463 | 0.099 | 5.720 | 0.025 | 3.910 | 0.098 |
| 336 | 1.503 | 0.107 | 5.908 | 0.038 | 3.931 | 0.073 |
| 337 | 1.529 | 0.110 | 5.679 | 0.012 | 3.714 | 0.048 |
| 338 | 1.503 | 0.104 | 6.103 | 0.051 | 4.060 | 0.110 |
| 339 | 1.574 | 0.124 | 6.049 | 0.021 | 3.843 | 0.050 |
| 340 | 1.530 | 0.112 | 6.479 | 0.021 | 4.235 | 0.081 |
| 341 | 1.549 | 0.117 | 5.756 | 0.050 | 3.717 | 0.089 |
| 342 | 1.492 | 0.100 | 5.932 | 0.015 | 3.975 | 0.105 |
| 343 | 1.497 | 0.100 | 6.611 | 0.013 | 4.416 | 0.102 |
| 344 | 1.433 | 0.097 | 5.704 | 0.021 | 3.979 | 0.098 |
| 345 | 1.320 | 0.096 | 6.376 | 0.038 | 4.829 | 0.324 |
| 346 | 1.472 | 0.101 | 6.389 | 0.016 | 4.341 | 0.061 |
| 347 | 1.492 | 0.108 | 6.370 | 0.023 | 4.270 | 0.115 |
| 348 | 1.411 | 0.100 | 6.519 | 0.022 | 4.621 | 0.178 |
| 349 | 1.412 | 0.081 | 6.130 | 0.037 | 4.340 | 0.123 |
| 350 | 1.531 | 0.118 | 4.431 | 0.013 | 2.894 | 0.062 |
| 351 |  |  | 0.000 |  |  |  |
| 352 |  |  | 0.000 |  |  |  |
| 353 |  |  | 0.000 |  |  |  |
| 354 | 1.462 | 0.097 | 6.090 | 0.026 | 4.164 | 0.088 |
| 355 | 1.428 | 0.095 | 6.406 | 0.016 | 4.485 | 0.129 |
| 356 | 1.510 | 0.108 | 6.799 | 0.019 | 4.501 | 0.086 |
| 357 | 1.455 | 0.094 | 6.804 | 0.032 | 4.678 | 0.102 |
| 358 | 1.504 | 0.119 | 6.710 | 0.014 | 4.460 | 0.166 |
| 359 | 1.449 | 0.091 | 5.710 | 0.054 | 3.941 | 0.163 |
| 360 | 1.477 | 0.106 | 6.780 | 0.013 | 4.590 | 0.144 |
| 361 | 1.354 | 0.066 | 6.706 | 0.025 | 4.955 | 0.146 |
| 362 | 1.341 | 0.079 | 7.140 | 0.087 | 5.325 | 0.270 |
| 363 | 1.511 | 0.116 | 4.654 | 0.032 | 3.080 | 0.111 |
| 364 | 1.417 | 0.082 | 5.470 | 0.014 | 3.861 | 0.079 |
| 365 | 1.466 | 0.107 | 5.323 | 0.037 | 3.632 | 0.133 |
| 366 | 1.508 | 0.123 | 5.910 | 0.020 | 3.919 | 0.153 |
| 367 | 1.460 | 0.092 | 4.922 | 0.057 | 3.370 | 0.101 |
| 368 | 1.590 | 0.126 | 5.052 | 0.008 | 3.177 | 0.068 |
| 369 | 1.552 | 0.116 | 5.811 | 0.040 | 3.743 | 0.148 |
| 370 | 1.493 | 0.096 | 5.743 | 0.014 | 3.847 | 0.138 |
| 371 | 1.447 | 0.092 | 6.002 | 0.070 | 4.148 | 0.101 |
| 372 | 1.680 | 0.156 | 4.592 | 0.032 | 2.733 | 0.071 |
| 373 | 1.399 | 0.075 | 5.236 | 0.031 | 3.742 | 0.104 |
| 374 |  |  | 0.000 |  |  |  |
| 375 | 1.485 | 0.099 | 5.573 | 0.030 | 3.754 | 0.072 |
| 376 | 1.559 | 0.119 | 4.813 | 0.026 | 3.087 | 0.083 |
| 377 | 1.562 | 0.120 | 4.935 | 0.015 | 3.159 | 0.078 |
| 378 | 1.598 | 0.128 | 4.860 | 0.021 | 3.041 | 0.064 |
| 379 | 1.558 | 0.138 | 4.450 | 0.017 | 2.856 | 0.116 |
| 380 | 1.730 | 0.212 | 4.018 | 0.026 | 2.323 | 0.394 |
| 381 | 1.552 | 0.121 | 4.300 | 0.048 | 2.771 | 0.113 |
| 382 | 1.548 | 0.129 | 3.694 | 0.030 | 2.387 | 0.103 |
| 383 | 1.548 | 0.126 | 4.601 | 0.039 | 2.973 | 0.115 |
| 384 | 1.520 | 0.119 | 5.049 | 0.029 | 3.322 | 0.087 |
| 385 | 1.681 | 0.154 | 4.231 | 0.011 | 2.518 | 0.054 |
| 386 | 1.643 | 0.138 | 4.177 | 0.019 | 2.542 | 0.046 |
| 387 | 1.559 | 0.120 | 2.991 | 0.009 | 1.918 | 0.036 |
| 388 |  |  | 0.000 |  |  |  |

| **Figure 2g** |  |  |
| --- | --- | --- |
| **Residue number** | **I_unsat_/I_sat_** | **Error** |
| 241 | -0.075 | 0.004 |
| 242 | 0.029 | 0.030 |
| 243 | 0.112 | 0.008 |
| 244 | 0.165 | 0.006 |
| 245 | 0.195 | 0.006 |
| 246 | 0.242 | 0.006 |
| 247 | 0.194 | 0.006 |
| 248 | 0.241 | 0.006 |
| 249 | 0.206 | 0.003 |
| 250 | 0.183 | 0.006 |
| 251 | 0.201 | 0.007 |
| 252 | 0.204 | 0.005 |
| 253 | 0.190 | 0.007 |
| 254 | 0.198 | 0.007 |
| 255 | 0.180 | 0.004 |
| 256 | 0.166 | 0.006 |
| 257 | 0.192 | 0.006 |
| 258 | 0.162 | 0.005 |
| 259 | 0.175 | 0.007 |
| 260 | 0.170 | 0.007 |
| 261 | 0.167 | 0.007 |
| 262 | 0.194 | 0.006 |
| 263 | 0.192 | 0.006 |
| 264 | 0.183 | 0.003 |
| 265 | 0.213 | 0.006 |
| 266 | 0.232 | 0.006 |
| 267 | 0.217 | 0.006 |
| 268 | 0.199 | 0.007 |
| 269 | 0.198 | 0.007 |
| 270 | 0.197 | 0.006 |
| 271 | 0.176 | 0.007 |
| 272 | 0.170 | 0.008 |
| 273 |  | 0.000 |
| 274 |  | 0.000 |
| 275 |  | 0.000 |
| 276 | 0.136 | 0.010 |
| 277 | 0.167 | 0.008 |
| 278 | 0.161 | 0.007 |
| 279 | 0.208 | 0.007 |
| 280 |  | 0.000 |
| 281 |  | 0.000 |
| 282 | 0.169 | 0.007 |
| 283 | 0.175 | 0.007 |
| 284 | 0.155 | 0.008 |
| 285 |  | 0.000 |
| 286 | 0.166 | 0.007 |
| 287 | 0.174 | 0.007 |
| 288 | 0.210 | 0.007 |
| 289 | 0.186 | 0.007 |
| 290 | 0.192 | 0.006 |
| 291 | 0.141 | 0.008 |
| 292 | 0.156 | 0.008 |
| 293 | 0.239 | 0.007 |
| 294 | 0.142 | 0.007 |
| 295 | 0.133 | 0.008 |
| 296 | 0.156 | 0.007 |
| 297 | 0.167 | 0.006 |
| 298 | 0.179 | 0.006 |
| 299 |  | 0.000 |
| 300 | 0.180 | 0.009 |
| 301 | 0.180 | 0.008 |
| 302 | 0.143 | 0.008 |
| 303 | 0.160 | 0.009 |
| 304 |  | 0.000 |
| 305 | 0.161 | 0.007 |
| 306 | 0.155 | 0.009 |
| 307 | 0.149 | 0.008 |
| 308 | 0.161 | 0.009 |
| 309 | 0.191 | 0.007 |
| 310 | 0.206 | 0.021 |
| 311 | 0.231 | 0.008 |
| 312 | 0.243 | 0.006 |
| 313 | 0.230 | 0.008 |
| 314 | 0.236 | 0.007 |
| 315 | 0.225 | 0.009 |
| 316 | 0.224 | 0.008 |
| 317 |  | 0.000 |
| 318 |  | 0.000 |
| 319 |  | 0.000 |
| 320 | 0.172 | 0.007 |
| 321 | 0.227 | 0.005 |
| 322 | 0.244 | 0.007 |
| 323 | 0.218 | 0.008 |
| 324 | 0.220 | 0.009 |
| 325 | 0.242 | 0.010 |
| 326 | 0.224 | 0.008 |
| 327 | 0.229 | 0.008 |
| 328 | 0.232 | 0.011 |
| 329 |  | 0.019 |
| 330 | 0.244 | 0.009 |
| 331 | 0.233 | 0.014 |
| 332 | 0.253 | 0.011 |
| 333 | 0.208 | 0.014 |
| 334 | 0.190 | 0.019 |
| 335 | 0.296 | 0.014 |
| 336 | 0.262 | 0.009 |
| 337 | 0.248 | 0.014 |
| 338 | 0.272 | 0.010 |
| 339 | 0.256 | 0.014 |
| 340 | 0.265 | 0.010 |
| 341 | 0.251 | 0.018 |
| 342 | 0.248 | 0.015 |
| 343 | 0.258 | 0.012 |
| 344 | 0.241 | 0.011 |
| 345 | 0.234 | 0.018 |
| 346 | 0.239 | 0.009 |
| 347 | 0.218 | 0.014 |
| 348 | 0.229 | 0.015 |
| 349 | 0.249 | 0.019 |
| 350 | 0.185 | 0.005 |
| 351 |  | 0.000 |
| 352 |  | 0.000 |
| 353 |  | 0.000 |
| 354 | 0.325 | 0.007 |
| 355 | 0.248 | 0.011 |
| 356 | 0.280 | 0.012 |
| 357 | 0.273 | 0.012 |
| 358 | 0.278 | 0.013 |
| 359 | 0.262 | 0.014 |
| 360 | 0.271 | 0.024 |
| 361 | 0.205 | 0.014 |
| 362 | 0.265 | 0.019 |
| 363 | 0.232 | 0.023 |
| 364 | 0.028 | 0.026 |
| 365 | 0.209 | 0.021 |
| 366 | 0.209 | 0.019 |
| 367 | 0.218 | 0.020 |
| 368 | 0.223 | 0.030 |
| 369 | 0.206 | 0.031 |
| 370 | 0.217 | 0.006 |
| 371 | 0.265 | 0.026 |
| 372 | 0.230 | 0.030 |
| 373 | 0.165 | 0.012 |
| 374 |  | 0.000 |
| 375 | 0.245 | 0.013 |
| 376 | 0.241 | 0.016 |
| 377 | 0.210 | 0.011 |
| 378 | 0.276 | 0.018 |
| 379 | 0.207 | 0.011 |
| 380 | 0.217 | 0.022 |
| 381 | 0.217 | 0.028 |
| 382 | 0.204 | 0.030 |
| 383 | 0.226 | 0.023 |
| 384 | 0.262 | 0.014 |
| 385 | 0.248 | 0.013 |
| 386 | 0.209 | 0.013 |
| 387 | 0.168 | 0.022 |
| 388 |  |  |
