# Supplementary material for: Multivalency regulates activity in an intrinsically disordered transcription factor
Source: eLife. 2018 May 1;7:e36258. doi: 10.7554/eLife.36258 (PMC5963919; doi:10.7554/eLife.36258)
Supplement: Figure 6—source data 1. — These values were used to create the graphs in Figure 6e–g. [file elife-36258-fig6-data1.docx]

|  | **dLBD I/I_0_, Averaged for each 10-amino acid motif** | | | | | | | |
| --- | --- | --- | --- | --- | --- | --- | --- | --- |
| **Molar Ratio** | **QT1** | **QT2** | **QT3** | **QT4** | **QT5** | **QT6** | **QT7** | **Control** |
| 1dLBD: 0 LC8 | 1.00 | 1.00 | 1.00 | 1.00 | 1.00 | 1.00 | 1.00 | 1.00 |
| 1:0.25 | 1.00 | 1.00 | 1.00 | 0.92 | 0.91 | 0.92 | 0.93 | 0.98 |
| 1:1 | 0.94 | 0.92 | 0.93 | 0.72 | 0.69 | 0.68 | 0.71 | 0.97 |
| 1:2 | 0.86 | 0.77 | 0.81 | 0.55 | 0.53 | 0.58 | 0.53 | 0.96 |
| 1:5 | 0.43 | 0.31 | 0.16 | 0.00 | 0.04 | 0.00 | 0.00 | 0.80 |
| 1:8 | 0.00 | 0.00 | 0.00 | 0.00 | 0.00 | 0.00 | 0.00 | 0.85 |

**Figure 6d,e**

**Figure 6f**

| **QT2-4 I/I_0_, Averaged for each 10-amino acid motif** | | | |
| --- | --- | --- | --- |
| **Molar Ratio** | **QT2** | **QT3** | **QT4** |
| 1 QT2-4: 0 LC8 | 1.00 | 1.00 | 1.00 |
| 1: 0.25 | 0.84 | 0.94 | 0.95 |
| 1:1 | 0.42 | 0.51 | 0.58 |
| 1:2 | 0.21 | 0.20 | 0.26 |
| 1:3 | 0.13 | 0.10 | 0.13 |
| 1:4 | 0.09 | 0.06 | 0.08 |

**Figure 6g**

| **QT4-6 I/I_0_, Averaged for each 10-amino acid motif** | | | |
| --- | --- | --- | --- |
| **Molar Ratio** | **QT4** | **QT5** | **QT6** |
| 1 QT4-6: 0 LC8 | 1.00 | 1.00 | 1.00 |
| 1: 0.25 | 0.85 | 0.83 | 0.78 |
| 1:1 | 0.18 | 0.26 | 0.18 |
| 1:2 | 0.04 | 0.08 | 0.05 |
| 1:3 | 0.02 | 0.04 | 0.02 |
| 1:4 | 0.01 | 0.02 | 0.01 |
