# Supplementary material for: Multivalency regulates activity in an intrinsically disordered transcription factor
Source: eLife. 2018 May 1;7:e36258. doi: 10.7554/eLife.36258 (PMC5963919; doi:10.7554/eLife.36258)
Supplement: Figure 7—source data 1. [file elife-36258-fig7-data1.docx]

| **Construct** | **pEGFP** | **ASCIZΔZnF** | **ASCIZ** | **AAA 8-11** | **AAA 1-4** | **AAA 5-11** | **AAA 1-4 8-11** | **AAA-all** |
| --- | --- | --- | --- | --- | --- | --- | --- | --- |
|  | 0.06 | 4.91 | 3.34 | 3.17 | 6.20 | 4.49 | 12.20 | 7.97 |
|  | 0.07 | 0.57 | 2.20 | 1.58 | 4.37 | 6.51 | 8.07 | 10.92 |
|  | 0.07 | 0.32 | 2.40 | 1.50 | 1.96 | 3.23 | 8.50 | 4.95 |
|  | 0.10 | 0.56 | 2.34 | 2.33 | 4.42 | 5.16 | 12.85 | 7.97 |
|  | 0.07 | 0.58 | 2.22 | 2.31 | 3.56 | 12.09 | 7.00 | 10.92 |
|  | 0.06 | 0.66 | 3.18 | 2.65 | 14.03 | 11.71 | 7.67 | 4.95 |
|  |  | 0.16 |  |  | 2.72 | 2.96 | 4.18 | 4.68 |
|  |  | 0.16 |  |  | 1.77 | 2.34 | 2.62 | 3.75 |
|  |  | 0.20 |  |  | 3.19 | 2.83 | 4.30 | 4.49 |
|  |  | 0.16 |  |  | 2.03 | 3.33 | 4.77 | 4.16 |
|  |  | 0.10 |  |  | 1.99 | 3.65 | 3.38 | 3.97 |
|  |  | 0.15 |  |  | 2.27 | 3.08 | 5.00 | 4.19 |
| **Average** | 0.07 | 0.71 | 2.61 | 2.26 | 4.04 | 5.12 | 6.71 | 6.08 |
| **Std Err** | 0.01 | 0.39 | 0.21 | 0.26 | 0.99 | 0.97 | 0.96 | 0.77 |
| **Kd** | N/A | N/A | 0.90 | 0.70 | 2.70 | 1.50 | 4.40 | 0.00 |
| **Kd Error** | N/A | N/A | 0.10 | 0.10 | 0.10 | 0.10 | 0.20 | 0.10 |
